# Strain characterization of multi-chamber cardiac dysfunction and associated prognosis in patients undergoing TAVR for severe AS

**DOI:** 10.1186/s44156-026-00122-6

**Published:** 2026-05-06

**Authors:** Thomas Meredith, David Roy, Farhan Mohammed, Amy Pomeroy, Michael P. Feneley, David W. M. Muller, Christopher Hayward, Mayooran Namasivayam

**Affiliations:** 1 Department of Cardiology, St Vincent’s Hospital, Level 4, Xavier Building, 438 Victoria Street, Darlinghurst, Sydney, NSW 2010 Australia; 2 Victor Chang Cardiac Research Institute, Sydney, Australia; 3 Faculty of Medicine and Health, University of New South Wales, Sydney, Australia

**Keywords:** Aortic stenosis, Strain imaging, TAVR, Echocardiography, Outcomes, Speckle tracking

## Abstract

**Background:**

In patients with severe aortic stenosis (AS) undergoing transcatheter aortic valve replacement (TAVR), myocardial dysfunction may extend beyond the left ventricle and remain underrecognized by conventional severity classification paradigms. Strain echocardiography allows early detection of subclinical dysfunction across cardiac chambers, potentially enhancing prognostic stratification.

**Methods:**

We retrospectively analysed 234 patients with severe AS undergoing TAVR who had echocardiograms suitable for strain analysis. Left ventricular (LV), left atrial (LA), and right ventricular (RV) strain values were quantified pre- and post-TAVR. Chamber dysfunction was defined using consensus strain thresholds, and multichamber impairment (MCI) was defined by dysfunction in two or more chambers. A modified damage staging system incorporating strain data was compared to an established model. The association between chamber impairment and all-cause mortality was explored.

**Results:**

Strain-defined chamber impairment was common, with 29% of patients exhibiting MCI. LA dysfunction was the most frequent isolated abnormality, accounting for 88% of single chamber impairment. Modest improvement was observed post-TAVR, predominantly in LV global longitudinal strain (+ 1.8 ± 3.5%, *p* < 0.01). MCI was associated with higher rates of atrial fibrillation, chronic kidney disease, and mitral/tricuspid regurgitation. At 12-month follow-up, patients with three-chamber impairment had significantly increased mortality risk (HR 6.84, 95% CI 1.77–26.4, *p* = 0.005). The modified staging system reclassified 27% of patients into higher risk categories but did not significantly improve predictive accuracy over the established model.

**Conclusion:**

Multichamber dysfunction, particularly involving the LA, is prevalent in patients undergoing TAVR and confers a higher early mortality risk. While the addition of strain data improves damage detection, its incremental prognostic value over conventional models appears modest in this cohort. Larger studies are required to explore this further. Comprehensive strain imaging may nonetheless identify patients requiring closer surveillance and targeted post-TAVR therapy.

**Supplementary Information:**

The online version contains supplementary material available at 10.1186/s44156-026-00122-6.

## Background

Aortic stenosis (AS) represents an increasing healthcare burden [1]. The current treatment paradigm, where valve replacement is indicated primarily for symptomatic severe AS quantified by haemodynamic criteria, likely underestimates the prevalence and impact of maladaptive cardiac dysfunction in this population. Evidence now demonstrates that cardiac damage to other chambers, beyond the left ventricle, is associated with poorer survival outcomes in AS patients [2].

Speckle tracking strain echocardiography has emerged as a powerful tool providing incremental diagnostic and prognostic information for a range of cardiomyopathic states, and is potentially a more sensitive marker of subtle cardiac chamber dysfunction [3]. Recent evidence suggests that comprehensive multi-chamber cardiac assessment enhances risk stratification in patients with AS [2]. Although the addition of left ventricular (LV) strain parameters to this staging framework adds incremental prognostic information [4], whether this is further optimised by the addition of lesser-utilised strain parameters, specifically right ventricular (RV) and left atrial strain, is the subject of ongoing research. Left atrial (LA) strain has gained recognition as an important marker of early diastolic dysfunction with independent prognostic information [5]. Specifically, LA reservoir strain (LASr)—a measure of atrial expansion during left ventricular systole—reflects LA compliance and function and can potentially signal dysfunction prior to gross atrial enlargement.

Accordingly, this study aimed to: (1) describe the distribution of LV, LA, and RV strain values in a contemporary cohort of patients with severe AS undergoing TAVR, and characterize any changes in these parameters post-procedure; (2) determine the incidence of chamber dysfunction as defined by established strain thresholds and compare these with cohort derived thresholds; (3) assess the impact of strain-define chamber impairment on survival; and (4) investigate any potential benefit of adding multichamber strain parameters to an established damage staging system.

## Methods

### Case selection

Our institutional database was screened for patients who had undergone transcatheter aortic valve replacement (TAVR) with locally available pre- and post-procedural echocardiograms of suitable image quality for analysis. At minimum, studies needed to include apical 2-, 3- and 4-chamber views to facilitate left ventricular chamber quantification speckle tracking. The database was screened sequentially from 2018 to June 2023. Cases were excluded if imaging was not performed on-site, or the indication for TAVR was not primary aortic stenosis (e.g. primary aortic regurgitation or valve-in-valve procedures). Baseline demographic, comorbidity and procedural data were abstracted from hospital medical records and clinic letters. Follow-up censoring occurred at last known interaction with state-based health services. The study was approved by the local institutional ethics board (St Vincent’s Hospital Human Research Ethics Committee ETH2021/11608).

### Strain analysis

Raw echocardiographic images were de-identified and re-analysed by an accredited Cardiologist or Cardiac Sonographer at the Heart Valve Disease and Artificial Intelligence Laboratory at the Victor Chang Cardiac Research Institute, Sydney. Conventional chamber measurements were performed according to contemporary ASE guidelines [6]. Strain analyses were performed with endocardial speckle-tracking using TomTec Arena software (TOMTEC Imaging Systems, Germany). In brief, semi-automated strain analysis was performed by automated detection of endocardial contours with manual adjustment by the operator if required. LV strain was measured using apical 2-, 3- and 4-chamber views. LA strain and RV free-wall strain was measured using the apical 4-chamber view. If available, the dedicated RV-focused view was used. Studies were only included for analysis if the chamber contours could be visualised and endocardial tracking was stable throughout the cardiac cycle.

### Cardiac chamber impairment

Chamber impairment was defined by contemporary strain thresholds which have demonstrated robust prognostic power in large trials or meta-analyses. Left ventricular impairment was defined by a LV GLS threshold of 15% [7, 8], which was also used recently in a similar strain based staging system reported by Tomaselli et al [9]. For the LA, we used LASr, which is rhythm independent and age- and sex-based thresholds (< 24% for males and < 21% for females over the age of 65) as proposed by Singh et al [10]. and further supported by Nagueh et al [11]. Multi-chamber impairment (MCI) was defined as the presence of more than one cardiac chamber which demonstrated a measured strain value below the currently accepted lower limit of normal. RV impairment was defined by the presence of RV free wall strain (FWS) < 20% [12, 13]. A modified cardiac damage staging system was generated based on the model proposed by Généreaux et al [2]. Details of the modified staging system are outlined in Supplemental Table 1. In brief, the criteria for LV, LA, and RV damage were extended to include strain-based determination of impairment of the corresponding chamber, as per the above thresholds.

### Statistical analysis

Continuous variables are presented as median and interquartile range and categorical variables as counts (n) and percentages. Absolute strain values are reported for simplicity. Between group comparisons were made with Wilcoxon rank sum test, Pearson’s Chi-squared test, or Fisher’s exact test, depending on variable type and normality distribution. Paired t-tests were utilised for pre- and post-TAVR strain comparisons. Kaplan–Meier curves were plotted for all-cause mortality and compared with the logrank test. Time to event analyses were performed with univariate and multivariate Cox proportional hazards modelling, including covariates of known prognostic significance in this population (age, sex, chronic kidney disease (CKD), Society of Thoracic Surgeons (STS) Score, atrial fibrillation, coronary artery disease). The proportional hazards assumption was assessed using Shoenfeld residuals. Multivariate Cox models were stratified by covariates which violated this assumption. To compare our dataset derived strain thresholds with those supported by the literature, receiver-operator characteristic curves were constructed for baseline chamber strain values, and optimal threshold predictive of all-cause mortality at longest follow up were identified using Youden’s index.

## Results

### Baseline characteristics

Screening identified 234 cases with adequate endocardial visualization to facilitate left ventricular strain assessment at minimum. Optimal right ventricular and left atrial endocardial tracking was present in 78% and 79% of the cohort, respectively. The mean age of the population was 81 years and 60% were male. The majority (84.3%) of patients underwent TAVR with a self-expanding prosthesis. The mean STS score was 5.8. Patients with multi-chamber impairment demonstrated a higher prevalence of AF, coronary artery disease and chronic kidney disease (CKD), but no difference in age, STS score, or other comorbid conditions. There were no significant differences in periprocedural outcomes, including requirement for implantable pacemaker. Baseline demographics and procedural characteristics are detailed in Table 1.

**Table 1:** Baseline characteristics

Characteristic	Overall *N* = 234^*1*^	One or Less *N* = 167^*1*^	More than One *N* = 67^*1*^	*p*-value^2^
Age	81 (10)	82 (11)	81 (7)	0.3
Sex				0.050
Female	93 (40%)	73 (44%)	20 (30%)	
Male	141 (60%)	94 (56%)	47 (70%)	
BMI	28.5 (20.8)	27.2 (4.7)	31.7 (38.3)	> 0.9
BSA (m^2^)	1.86 (0.22)	1.85 (0.21)	1.88 (0.24)	0.2
Hypertension	143 (64%)	99 (63%)	44 (66%)	0.7
Diabetes	56 (25%)	35 (22%)	21 (31%)	0.14
Hyperlipidaemia	149 (76%)	105 (78%)	44 (72%)	0.4
Coronary artery disease	113 (50%)	70 (44%)	43 (64%)	**0.006**
Previous Stroke/TIA	20 (8.9%)	14 (8.9%)	6 (9.0%)	> 0.9
Atrial Fib/Flutter	63 (28%)	35 (22%)	28 (42%)	**0.002**
Active Smoker	6 (2.7%)	5 (3.2%)	1 (1.5%)	0.7
Severe lung disease	37 (20%)	29 (23%)	8 (14%)	0.14
Sleep Apnoea	19 (10%)	14 (11%)	5 (8.5%)	0.6
Chronic kidney disease	97 (43%)	58 (37%)	39 (58%)	**0.003**
Severe peripheral arterial disease	20 (11%)	15 (12%)	5 (8.3%)	0.4
NYHA Score				0.064
1	7 (3.2%)	5 (3.2%)	2 (3.0%)	
2	162 (73%)	120 (78%)	42 (63%)	
3	39 (18%)	23 (15%)	16 (24%)	
4	13 (5.9%)	6 (3.9%)	7 (10%)	
Serum creatinine	95 (76, 119)	89 (71, 116)	97 (82, 129)	**0.025**
eGFR	59 (20)	61 (20)	57 (20)	0.2
Serum albumin	35.7 (4.4)	36.1 (4.4)	35.0 (4.4)	0.3
NT-proBNP	2,358 (742, 4,026)	2,358 (731, 5,666)	2,358 (934, 3,840)	> 0.9
Hb	125 (18)	124 (18)	126 (18)	0.6
RDW	15.09 (2.15)	14.90 (1.91)	15.50 (2.58)	0.059
Platelets	204 (67)	204 (64)	203 (73)	0.7
WCC	7.01 (2.67)	7.04 (2.59)	6.93 (2.87)	0.15
STS Score	5.8 (4.5)	5.4 (3.3)	6.7 (6.5)	> 0.9
Risk Category (STS)				0.7
High	41 (20%)	27 (18%)	14 (23%)	
Intermediate	83 (40%)	61 (42%)	22 (35%)	
Low	84 (40%)	58 (40%)	26 (42%)	
Valve Type				**0.034**
Sapien 3	26 (11%)	14 (8.4%)	12 (18%)	
Sapien 3 Ultra	12 (5.1%)	10 (6.0%)	2 (3.0%)	
Evolut-R	151 (65%)	105 (63%)	46 (69%)	
Evolut-Pro	35 (15%)	28 (17%)	7 (10%)	
Portico	10 (4.3%)	10 (6.0%)	0 (0%)	
Fluoroscopic PVL				0.3
None	29 (15%)	24 (18%)	5 (8.8%)	
Trivial/Mild	160 (83%)	108 (79%)	52 (91%)	
Moderate	2 (1.0%)	2 (1.5%)	0 (0%)	
Severe	2 (1.0%)	2 (1.5%)	0 (0%)	
Complications				
Coronary obstruction	0 (0%)	0 (0%)	0 (0%)	> 0.9
Conversion to sternotomy	1 (0.5%)	1 (0.7%)	0 (0%)	> 0.9
Device embolisation	4 (1.9%)	2 (1.3%)	2 (3.2%)	0.6
Second valve	3 (1.4%)	1 (0.7%)	2 (3.2%)	0.2
Annular rupture	0 (0%)	0 (0%)	0 (0%)	> 0.9
Haemodynamic collapse	3 (1.4%)	2 (1.3%)	1 (1.6%)	> 0.9
MCS	1 (0.5%)	0 (0%)	1 (1.6%)	0.3
Vascular complication	2 (0.9%)	1 (0.6%)	1 (1.5%)	0.5
Procedural stroke	5 (2.3%)	4 (2.6%)	1 (1.6%)	> 0.9
Need for PPM	15 (7.0%)	11 (7.3%)	4 (6.3%)	> 0.9

^*1*^ Mean (SD); n (%); Median (Q1, Q3); BMI = body mass index; BSA = body surface area; MCS = mechanical circulatory support; PPM = permanent pacemaker; PVL = paravalvular leak; RDW = red cell distribution width; WCC = white cell count

^*2*^ Wilcoxon rank sum test; Pearson’s Chi-squared test; Fisher’s exact test

### Echocardiographic characteristics

**Fig. 1:**
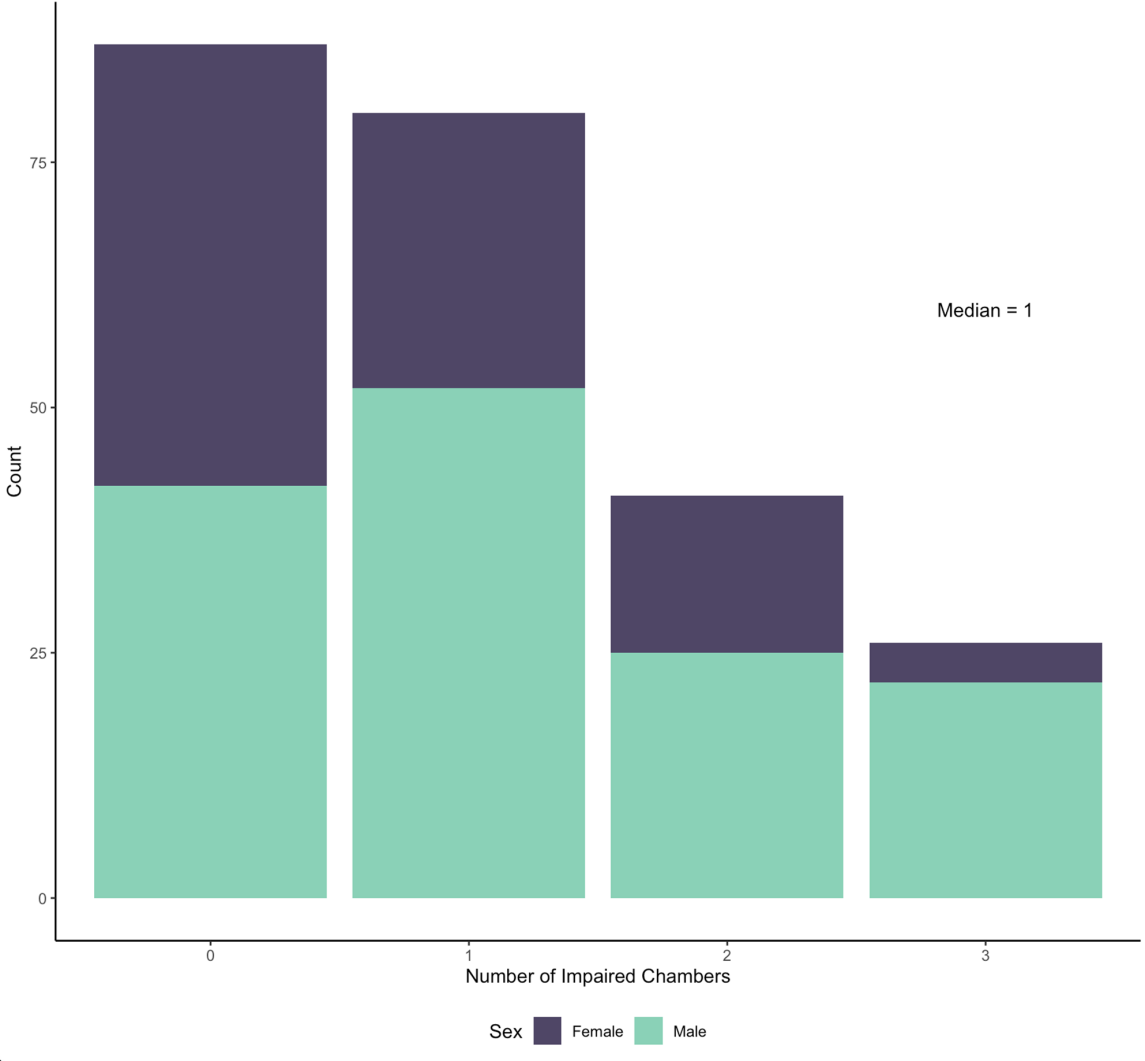
Histogram of chamber impairment across the cohort, stratified by sex

Echocardiograms were performed at a median of 25 days prior to TAVR and a median of 1 days after. All patients demonstrated severe aortic stenosis by echocardiographic criteria; the mean pre-procedural AVA was 0.91 ± 0.33cm^2^, peak gradient of 62 ± 21mmHg, mean gradient 38 ± 13 mmHg, and dimensionless index (DI) of 0.26 ± 0.09. The cohort included patients with low-flow low-gradient AS, representing 16% (*n* = 35). Most patients demonstrated impairment in at least one cardiac chamber (median 1), as quantified by strain (Fig. 1).

Patients with MCI had a discordant trend towards more severe valve stenosis, with a non-significant difference in AVA (0.89 vs. 0.91, *p* = 0.6) but significant difference in dimensionless index (DI, 0.24 vs. 0.27, *p* < 0.01). Patients with MCI demonstrated worse overall indices of cardiac chamber function and had a higher prevalence of greater than mild tricuspid and mitral valve regurgitation but no difference in aortic regurgitation (Table 2).

**Table 2:** Baseline echocardiographic characteristics

Characteristic	Overall *N* = 234^*1*^	One or Less *N* = 167^*1*^	More than One *N* = 67^*1*^	*p*-value^2^
**Valve Indices**				
**AVA**	0.91 (0.33)	0.91 (0.33)	0.89 (0.35)	0.6
**DTI**	0.26 (0.09)	0.27 (0.09)	0.24 (0.10)	**< 0.001**
**AV Mean Gradient**	38 (13)	39 (14)	36 (13)	0.2
**AV Peak Gradient**	62 (21)	64 (21)	58 (19)	0.12
**Peak Velocity**	3.89 (0.65)	3.94 (0.65)	3.77 (0.64)	0.12
**SVi**	43(15)	45(15)	39 (13)	**0.001**
**Aortic Regurgitation**				0.086
None/Trace	73 (31%)	53 (32%)	20 (30%)	
Mild	123 (53%)	83 (50%)	40 (60%)	
Moderate	31 (13%)	27 (16%)	4 (6.0%)	
Severe	6 (2.6%)	3 (1.8%)	3 (4.5%)	
**Mitral Regurgitation**				**0.008**
None/Trace	34 (15%)	28 (17%)	6 (9.0%)	
Mild	160 (68%)	118 (71%)	42 (63%)	
Moderate	35 (15%)	20 (12%)	15 (22%)	
Severe	5 (2.1%)	1 (0.6%)	4 (6.0%)	
**Tricuspid regurgitation**				**0.005**
None/Trace	57 (24%)	45 (27%)	12 (18%)	
Mild	149 (64%)	110 (66%)	39 (58%)	
Moderate	23 (9.8%)	10 (6.0%)	13 (19%)	
Severe	5 (2.1%)	2 (1.2%)	3 (4.5%)	
**LV Indices**				
**IVSd (mm)**	12.82 (2.26)	12.84 (2.21)	12.77 (2.39)	0.8
**PWD (mm)**	12.41 (2.09)	12.41 (2.13)	12.43 (1.98)	0.5
**LV GLS %**	-17.1 (4.5)	-19.3 (2.9)	-12.5 (3.7)	**< 0.001**
**LVEF %**	58 (13)	63 (9)	47 (14)	**< 0.001**
**LA Indices**				
**LAVi**	48 (15)	44 (13)	57 (16)	**< 0.001**
**Reservoir Strain %**	18 (9)	21 (9)	12 (5)	**< 0.001**
**Booster Strain %** ^**3**^	-10.4 (6.3)	-12.0 (6.1)	-6.5 (4.8)	**< 0.001**
**Conduit Strain %**	-6 (8)	-8 (8)	-2 (8)	**< 0.001**
**RV Indices**				
**RV FWS %**	-24 (6)	-27 (5)	-19 (6)	**< 0.001**
**RV GLS %**	-19.6 (5.6)	-22.1 (4.3)	-15.1 (4.7)	**< 0.001**
**Basal Diameter (mm)**	38.2 (5.7)	37.4 (5.5)	39.9 (6.0)	**0.009**
**TAPSE (mm)**	21.9 (4.6)	22.9 (4.4)	19.8 (4.2)	**0.004**
**RV S’**	13.2 (3.3)	13.5 (2.7)	12.7 (4.2)	0.9
**RVSP (mmHg)**	30 (12)	29 (11)	31 (13)	0.2

^1^ Mean (SD); n (%)

^2^ Wilcoxon rank sum test; Pearson’s Chi-squared test

^3^Booster/conduit strain measured only when sinus rhythm present (*n* = 156)

**Fig. 2:**
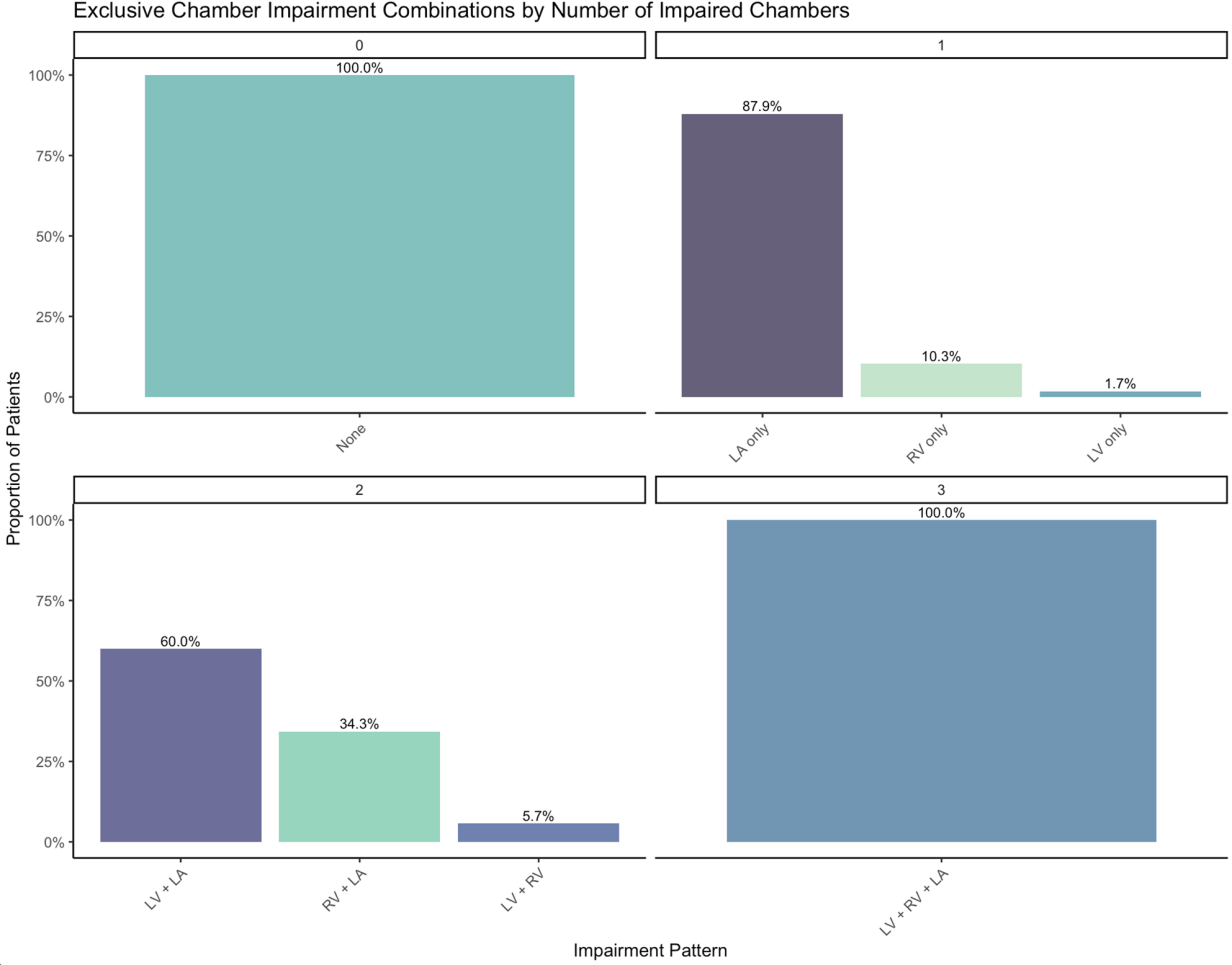
Bar charts grouped by chamber and number of chambers impaired. Single chamber impairment is dominated by isolated left atrial dysfunction, representing 88% of cases. Isolated LV impairment was identified in 1.7% of cases

In patients with any chamber impairment, single chamber impairment was the most common entity, predominantly driven by LA impairment which accounted for 88% of instances of single chamber impairment (Fig. 2). Isolated RV impairment was rare (10.3%), as was LV impairment in the absence of LA impairment (1.7%). From our dataset, predictive thresholds for LV GLS, LASr, and RV FWS were 16.78% (vs. consensus 15%), 19% (vs. 24/21%), and 17.4% (vs. 20%), respectively.

### Chamber function following TAVR

**Fig. 3:**
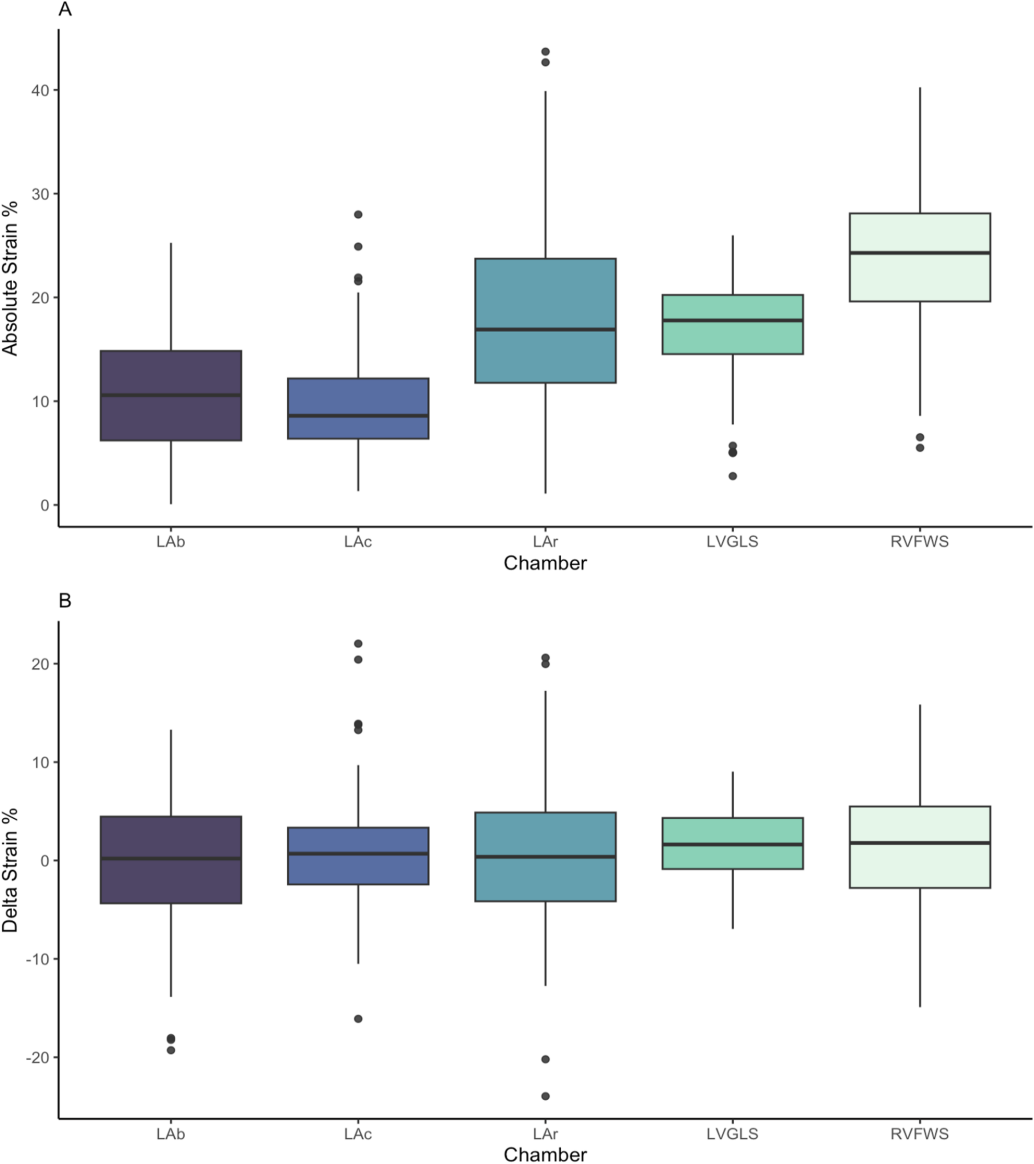
Panel A = distribution of baseline chamber strain values; Panel B = distribution of absolute change in chamber strain values post-TAVR

**Fig. 4:**
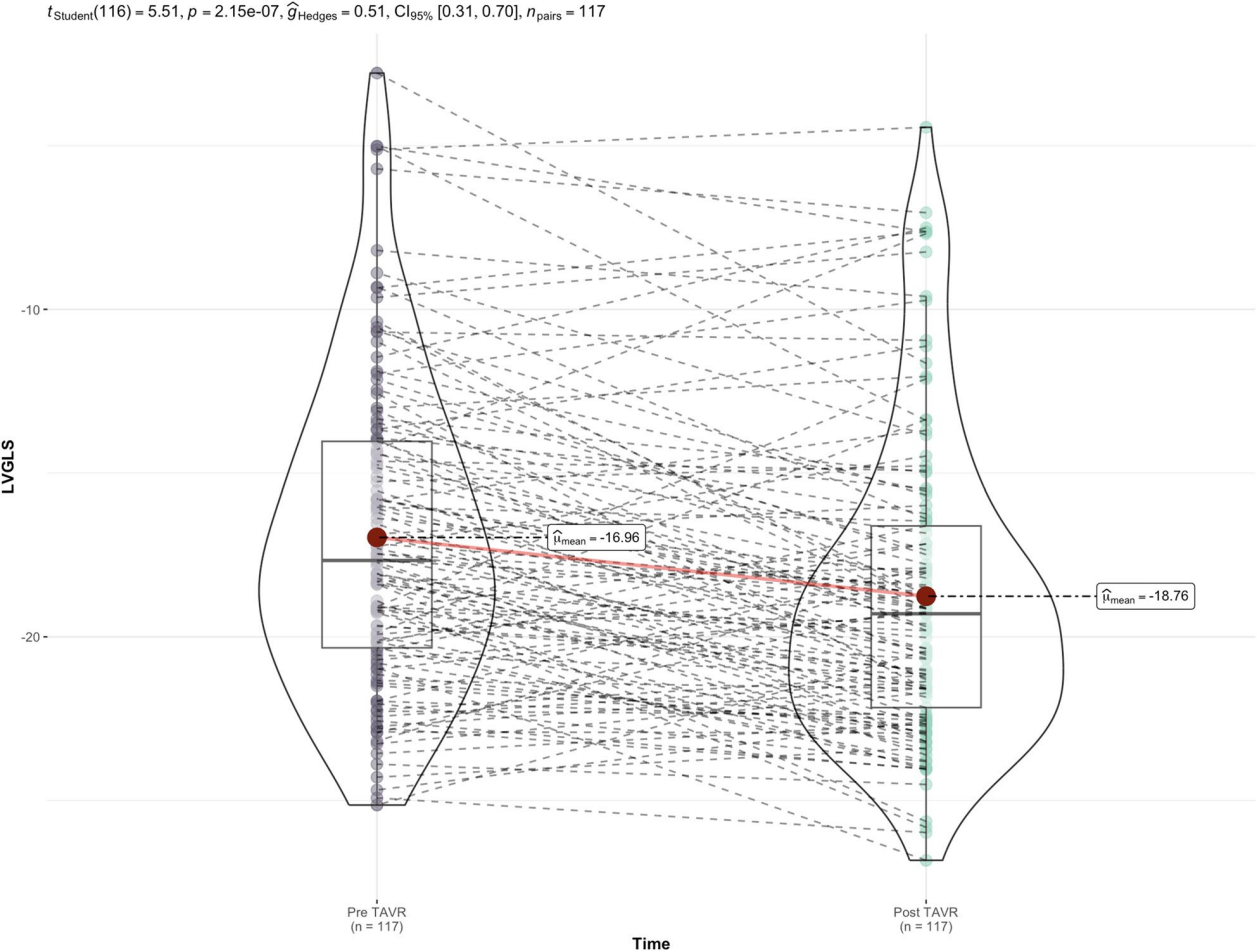
Pairwise violin plots demonstrating a significant change in LVGLS from baseline (left) to post-TAVR (right)

The mean cohort change in LVGLS was + 1.8 ± 3.53%, RVFWS + 1.24 ± 6.69%, and LAS_R_ +0.9 ± 7.61% (Fig. 3). Patients with MCI demonstrated numerical non-significant trends towards greater improvement in LVGLS and RVFWS (Table 3). In global pairwise analysis, only LVGLS significantly changed post-TAVR (Fig. 4).

**Table 3:** Comparison of mean absolute changes in chamber strain values between groups with and without multichamber impairment

Characteristic	Overall *N* = 234^*1*^	One or Less *N* = 167^*1*^	More than One *N* = 67^*1*^	*p*-value^2^
LVGLS	1.8 (3.5)	1.3 (3.2)	2.7 (3.9)	0.060
RVFWS	1 (7)	0 (6)	3 (7)	0.055
LASr	1 (8)	0 (8)	2 (7)	0.3
LASb	-0.1 (6.4)	-0.9 (7.0)	1.3 (4.9)	0.2
LASc	1.0 (5.5)	1.1 (5.9)	0.9 (4.9)	> 0.9

^1^ Mean (SD)

^2^ Wilcoxon rank sum test

### Damage stage reclassification

The addition of multichamber strain criteria to the damage staging system proposed by Généreaux et al. resulted in the reclassification of 63 patients into high (worse) grades, and none into lower grades (Supplemental Figure S1).

### Outcomes

**Fig. 5:**
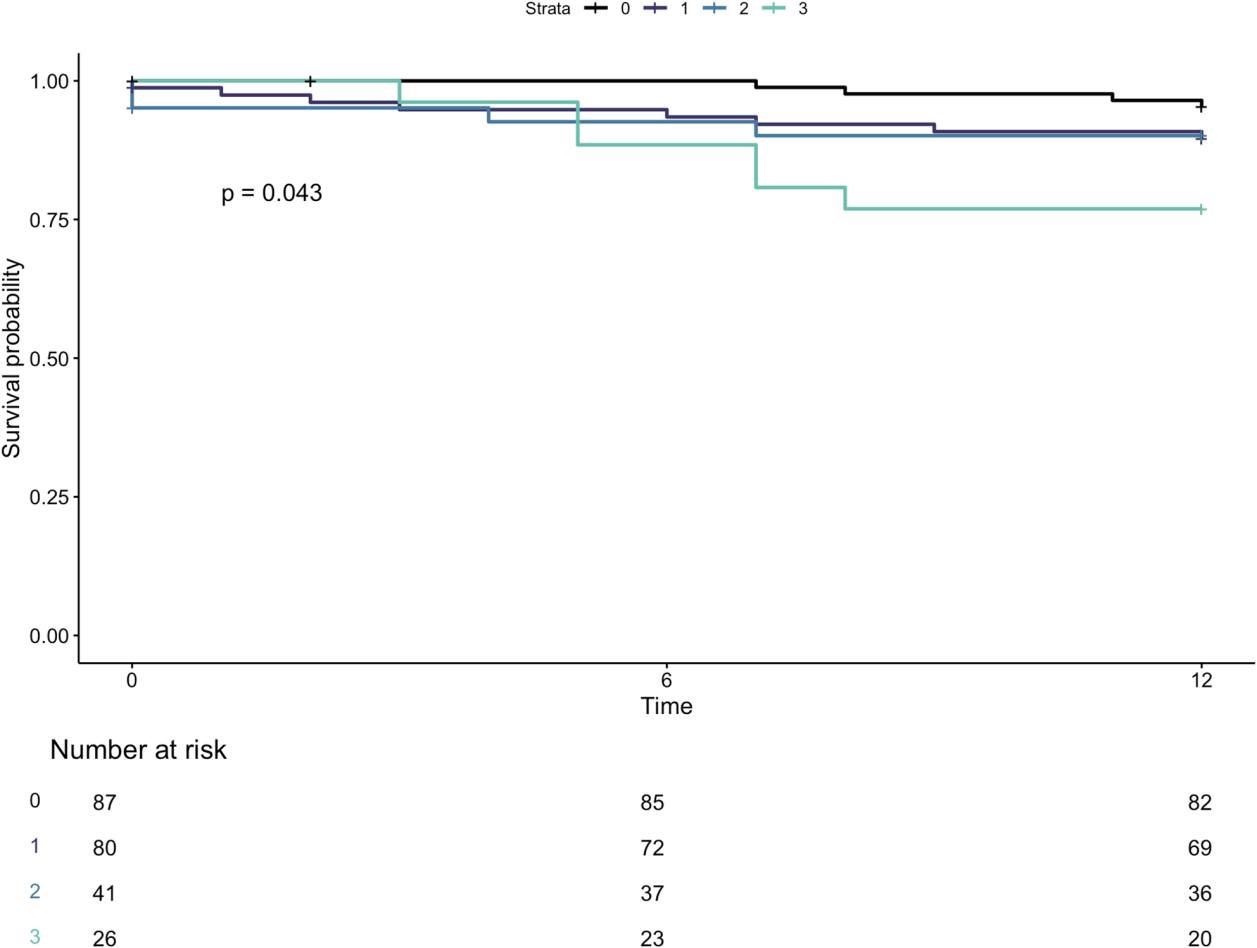
Kaplan-Meier curves demonstrating 12-month survival of patients grouped by number of impaired chambers, as quantified by respective strain thresholds

**Fig. 6:**
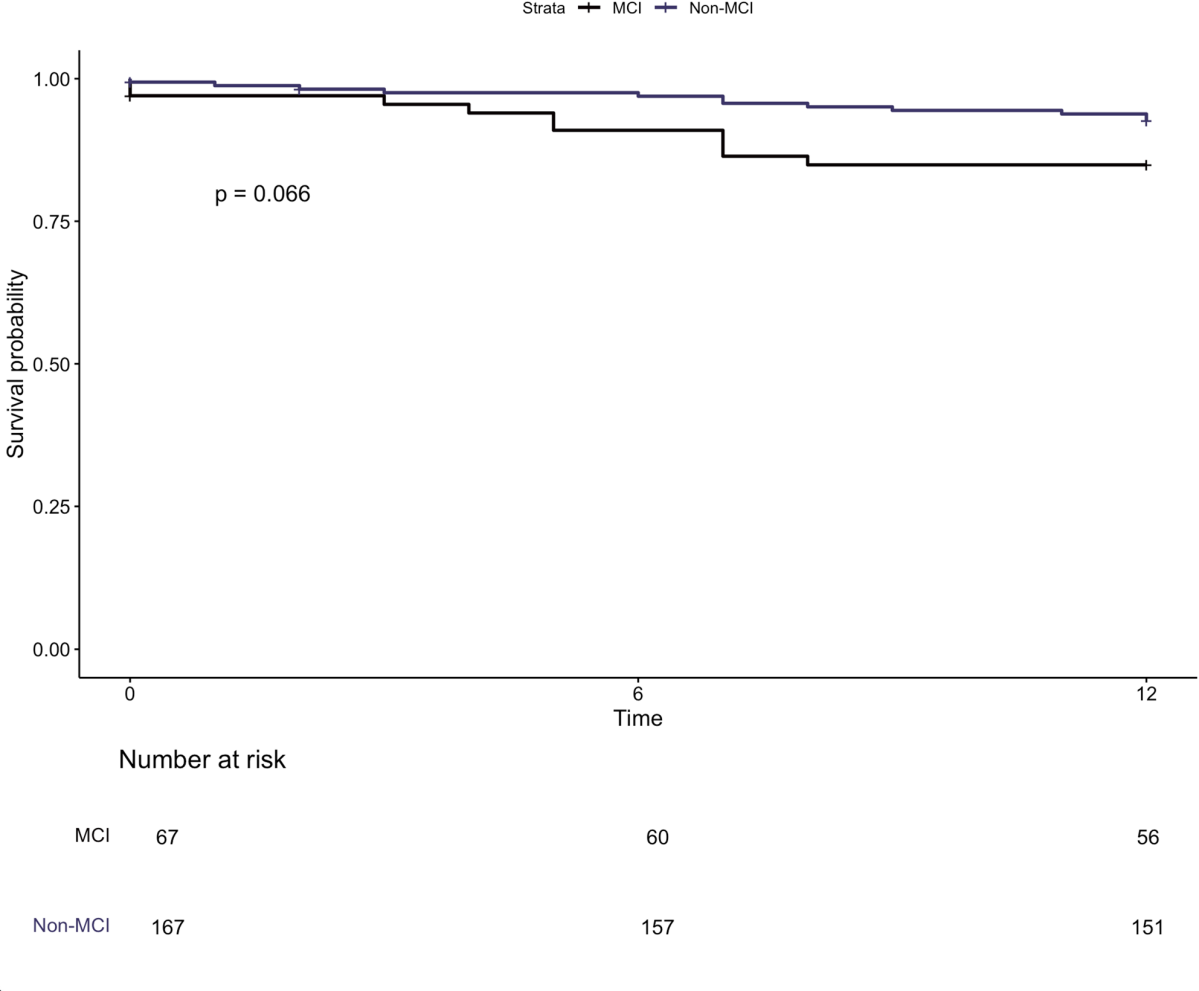
Kaplan-Meier curves demonstrating 12-months survival of patients with and without multichamber impairment (MCI)

The cohort median survival was 59 months with a median follow up 28 months. At 12-month landmark analysis, there was progressive deterioration in survival with increasing number of chambers impaired at baseline (Fig. 5, logrank *p* = 0.04). Compared to those without any chamber impairment, patients with three chambers impaired experienced a 5-fold increased risk of 12-month mortality (HR5.49, 95% CI 1.5-19.469, *p* < 0.01), on both univariate and multivariate analyses (Table 4). This trend continued at maximum follow up (Supplemental Figure S2). When categorised by the presence of multi-chamber dysfunction, Patients with MCI demonstrated a non-significant trend to worse survival at 12-month landmark analysis (Fig. 6, logrank 0.07), followed by near convergence of curves at 24 months (Supplemental Figure S3). At maximum follow-up, there was no significant difference between groups. The addition of strain criteria to the established damage staging system neither improved nor diminished survival prediction at maximum follow up (AIC 578.6 vs. 581.3 for established and modified systems, respectively). Patients classified as modified stage 4 demonstrated significantly worse mortality compared with those in stage 0 (HR 3.0, 95% CI 1.0-8.9, *p* < 0.05). Derived chamber-specific strain thresholds predictive of 12-month mortality are outlined in Supplemental Table 2.

**Table 4:** Univariate and multivariate cox proportional hazards models analysing the influence of chamber impairment on 12-month mortality, together with other clinically important covariates known to be associated with adverse outcome. Multivariate analysis stratified by CKD owing to violation of proportional hazards

Characteristic	Univariate				Multivariate		
	*N*	HR	95% CI	*p*-value	HR	95% CI	*p*-value
Number of impaired chambers	234	—	—				
1		2.35	0.71, 7.79	0.2	2.93	0.85, 10.0	0.088
2		2.27	0.57, 9.08	0.2	2.11	0.48, 9.30	0.3
3		5.49	1.55, 19.5	**0.008**	6.84	1.77, 26.4	**0.005**
Age	233	1.00	0.95, 1.04	0.9	0.99	0.96, 1.02	0.6
STS Score	208	1.11	1.05, 1.18	**< 0.001**	1.11	1.02, 1.20	**0.011**
Male vs. Female	234	0.65	0.28, 1.51	0.3	0.80	0.30, 2.16	0.7
CKD	225	3.59	1.41, 9.18	**0.008**			
CAD	225	0.67	0.29, 1.57	0.4	0.43	0.16, 1.12	0.082
AF	226	1.25	0.51, 3.05	0.6	0.89	0.34, 2.37	0.8

Abbreviations: AF = atrial fibrillation; CAD = coronary artery disease; CI = Confidence Interval, HR = Hazard Ratio

## Discussion

The present study provides several important insights regarding cardiac chamber function, as quantified by echocardiographic strain imaging, in patients undergoing TAVR for severe aortic stenosis. Notable findings include the high prevalence of strain-determined MCI in this contemporary TAVR cohort, occurring in 29% of patients. The presence of MCI is associated with a non-significant trend towards worse survival at 12 months post TAVR. While the addition of strain criteria to a contemporary damage staging system provided greater sensitivity for damage, this was not associated with a meaningful improvement in predictive power in this single-centre cohort. Additionally, while some degree of early functional recovery across chambers is observed following TAVR, this recovery is modest and predominantly limited to the left ventricle.

Left atrial dysfunction was the most common manifestation of single-chamber impairment, accounting for 88% of these cases. This interesting observation supports the pathophysiological sequence in pressure-overload states, where increased LV filling pressures directly impact atrial mechanics before overt ventricular dysfunction becomes apparent [14, 15]. The rarity of isolated right ventricular (9%) or left ventricular (4%) dysfunction without concurrent atrial impairment further supports this. This proposed sequence lies in contrast to the established damage staging system by Genereaux et al., in which LV damage (defined by the presence of an ejection fraction < 50%) exists at an earlier and prognostically more favourable stage (Stage 1), compared with the presence of LA damage (Stage 2). Further research is required to elucidate the prevalence and predictive utility of impaired left atrial strain for in early stages of aortic stenosis.

Patients with multichamber impairment exhibited several distinguishing features compared to those with single or no chamber dysfunction. These included higher prevalence of coronary artery disease, atrial fibrillation, and chronic kidney disease—all factors known to independently affect myocardial performance and strain parameters [16, 17]. The observed discordance between calculated aortic valve area and dimensionless index in patients with multichamber dysfunction also likely reflects more substantial myocardial remodelling and contractile inefficiency in these patients, associated with reduced stroke volume index.

### Chamber function post-TAVR

The observed improvement in LVGLS (+ 1.8 ± 3.53%), though modest, was statistically significant in pairwise analysis, consistent with previous studies demonstrating partial recovery of LV mechanics following TAVR [18–23]. The magnitude of improvement was numerically greater in patients with multichamber impairment (+ 2.7% vs. + 1.3%), suggesting potentially greater reversibility in this subgroup, though these differences did not reach statistical significance. This pattern of improvement is consistent with previous investigations showing that patients with more impaired baseline function may demonstrate greater absolute improvement following relief of pressure overload, potentially reflecting a relatively greater contribution reversible afterload mismatch rather than pure irreversible myocardial damage [21, 24]. Our observed trend towards greater improvement in those with more severe baseline impairment, albeit with substantial variance, supports this hypothesis, however further research is needed to delineate any prognostic implications beyond these exploratory observations.

### Prognostic implications

An important finding of this study is the significant association between chamber impairment and mortality. Compared to patients without any chamber impairment, patients with three-chamber impairment experienced a 5-fold higher risk of 12-month mortality. This relationship persisted even after adjustment for multiple clinically relevant variables known to be associated with adverse outcome, including STS Score, suggesting that strain-defined chamber dysfunction provides incremental prognostic information beyond conventional risk metrics. The convergence of survival curves at approximately 24 months is also interesting. This pattern suggests that multichamber impairment may be particularly predictive of early post-procedural outcomes, while longer-term survival is likely influenced by additional factors including age and associated comorbidities. The addition of strain-based criteria to the established damage staging system neither improved nor diminished survival prediction at maximum follow-up suggesting that conventional parameters of chamber structure and function already capture the bulk of important prognostic detail. Nevertheless, patients classified as modified stage 4 demonstrated significantly worse mortality compared with those in stage 0 (HR 3.0, 95% CI 1.0-8.9, *p* < 0.05), indicating that the most advanced forms of chamber dysfunction identified by strain may still provide incremental prognostic value in selected patients.

### Limitations

Several limitations of our study warrant consideration. The single-centre retrospective design and modest sample size limit the generalizability of our findings and preclude definitive conclusions regarding causality. Additionally, our cohort of patients undergoing TAVR naturally includes patients at the end-stage of the disease spectrum, and multichamber assessment may demonstrate incremental prognostic utility in earlier disease stages. The variability in timing of post-TAVR echocardiographic assessment may have influenced our assessment of chamber recovery, particularly given the potential for early versus late remodelling patterns, which have been well described. Additionally, The technical challenge in obtaining LA and RV strain may also reduce the sensitivity in detecting chamber impairment for the cohort of patients with suboptimal image quality. Comprehensive three-chamber strain analysis was not feasible in all patients, particularly for LA and RV strain, which are more dependent on optimal chamber-specific image acquisition. This may have led to underestimation of multichamber impairment and limits direct generalizability to cohorts undergoing standardized prospective strain imaging. Finally, our analysis did not account for potential confounding from several known and unknown variables which can influence baseline strain values as well as strain recovery, such as coexisting coronary or pulmonary disease, or post-procedural medical therapy. While we adjusted for numerous potential confounders in our survival analyses, residual confounding by unmeasured variables cannot be excluded.

### Future directions

Larger, prospective studies with standardized timing of echocardiographic assessment and longer follow-up are needed to validate the prognostic utility of multichamber strain imaging in TAVR patients, particularly the significance of LA dysfunction. The relationship between strain patterns and functional outcomes, including exercise capacity, quality of life, and heart failure hospitalizations, also warrants further exploration, as these endpoints may be more sensitive to changes in chamber function than all-cause mortality. The potential utility of artificial intelligence and machine learning approaches to integrate multiple strain parameters into a unified risk prediction model represents a promising avenue for further research, particularly given the complex interactions between chambers and the potential for compensatory mechanisms that may not be captured by analysis of individual chamber function. Finally, interventional studies evaluating whether targeted therapies to address persistent chamber dysfunction can improve post-TAVR outcomes would be of considerable clinical interest. For example, investigating whether early institution of neurohormonal antagonists in patients with persistent multichamber dysfunction after TAVR improves strain recovery and clinical outcomes could provide valuable insights into the optimal management of this high-risk subgroup.

## Conclusion

Multichamber dysfunction as defined by strain imaging is common in patients undergoing TAVR for severe aortic stenosis and is associated with increased mortality, particularly within the first year following the procedure. While TAVR results in modest improvement in left ventricular mechanics, upstream chamber dysfunction often persists, suggesting a more advanced and potentially less reversible stage of cardiac remodelling. Comprehensive strain assessment may enhance risk stratification in this population and identify patients who might benefit from more intensive post-procedural monitoring and management.

## Supplementary Information

Below is the link to the electronic supplementary material.

Supplementary Material 1

## Data Availability

The data are available upon request and with the permission from the authors.
