# Supplementary material for: Strain characterization of multi-chamber cardiac dysfunction and associated prognosis in patients undergoing TAVR for severe AS
Source: Echo Res Pract. 2026 May 6;13:13. doi: 10.1186/s44156-026-00122-6 (PMC13147892; doi:10.1186/s44156-026-00122-6)
Supplement: Supplementary file 1 — Supplementary Material 1 [file 44156_2026_122_MOESM1_ESM.docx]

**Supplemental Material**

Strain Characterization of Multi-Chamber Cardiac Dysfunction and Associated Prognosis in Patients Undergoing TAVR for Severe AS

**Supplemental Tables**

| Damage Stage | Généreaux Definition | Modified Definition |
| --- | --- | --- |
| 0 | No other cardiac damage detected | Unchanged |
| 1 | LV damage as defined by presence of LV hypertrophy (LV mass index >95 g/m^2^ for women, >115 g/m^2^ for men), severe LV diastolic dysfunction (E/e′ > 14), or LV systolic dysfunction (LV ejection fraction <50%) | Généreaux criteria + presence of LV GLS <15% |
| 2 | LA or mitral valve damage or dysfunction as defined by the presence of an enlarged left atrium (>34 mL/m^2^), the presence of atrial fibrillation, or the presence of moderate or severe mitral regurgitation | Généreaux criteria + presence of LASr <24% for males and <21% for females |
| 3 | Pulmonary artery vasculature or tricuspid valve damage or dysfunction as defined by the presence of systolic pulmonary hypertension (systolic pulmonary arterial pressure ≥60 mmHg) or the presence of moderate or severe tricuspid regurgitation | Unchanged |
| 4 | RV damage as defined by the presence of moderate or severe RV dysfunction | Généreaux criteria + presence of RV FWS <20% |

Table S1: Modified staging system criteria

| Stain Variable | Predictive Threshold |
| --- | --- |
| LA Reservoir | 19.02 |
| LV GLS | 16.78 |
| RV FWS | 17.4 |

Table S2: Chamber-specific strain thresholds predictive of all-cause mortality at longest follow up identified using Youden’s index. FWS = free wall strain; GLS = global longitudinal strain.

**Supplemental Figures**


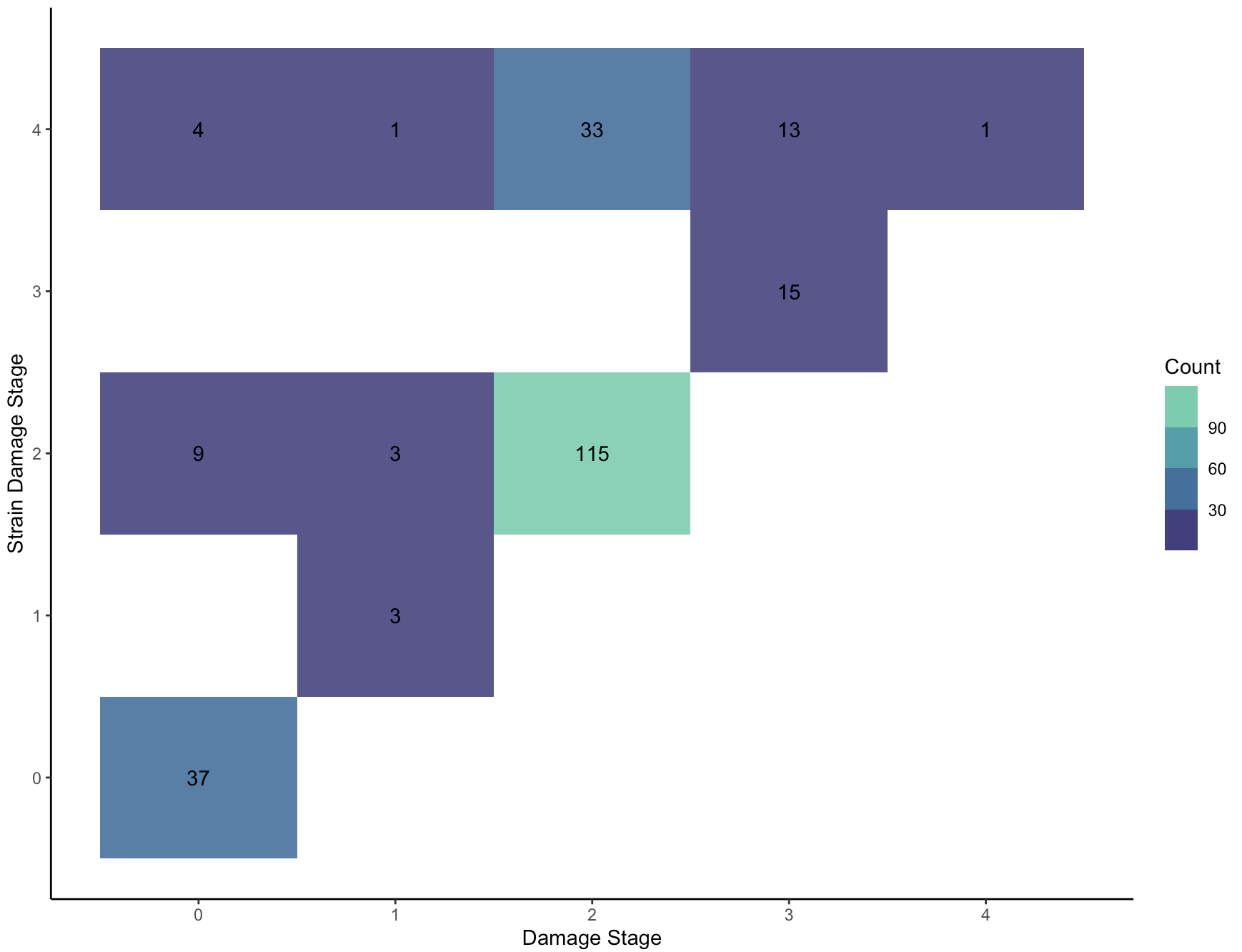


Figure S1: Tile plot demonstrating reclassification of patients from the established damage staging (x-axis) to a modified strain-based damage system (y-axis).


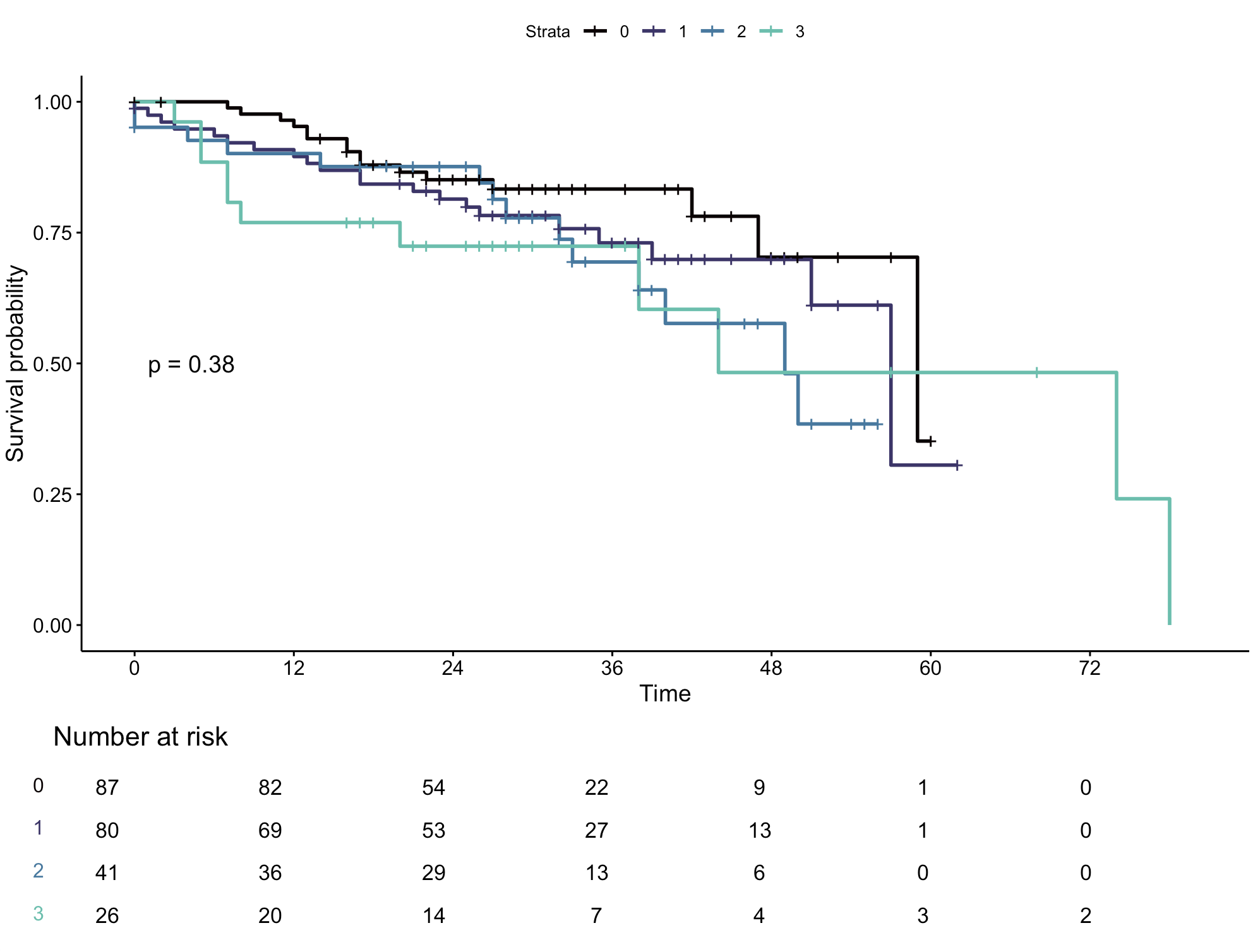


Figure S2: Kaplan-Meier curves for patients grouped by number of chambers impaired, at longest follow up.


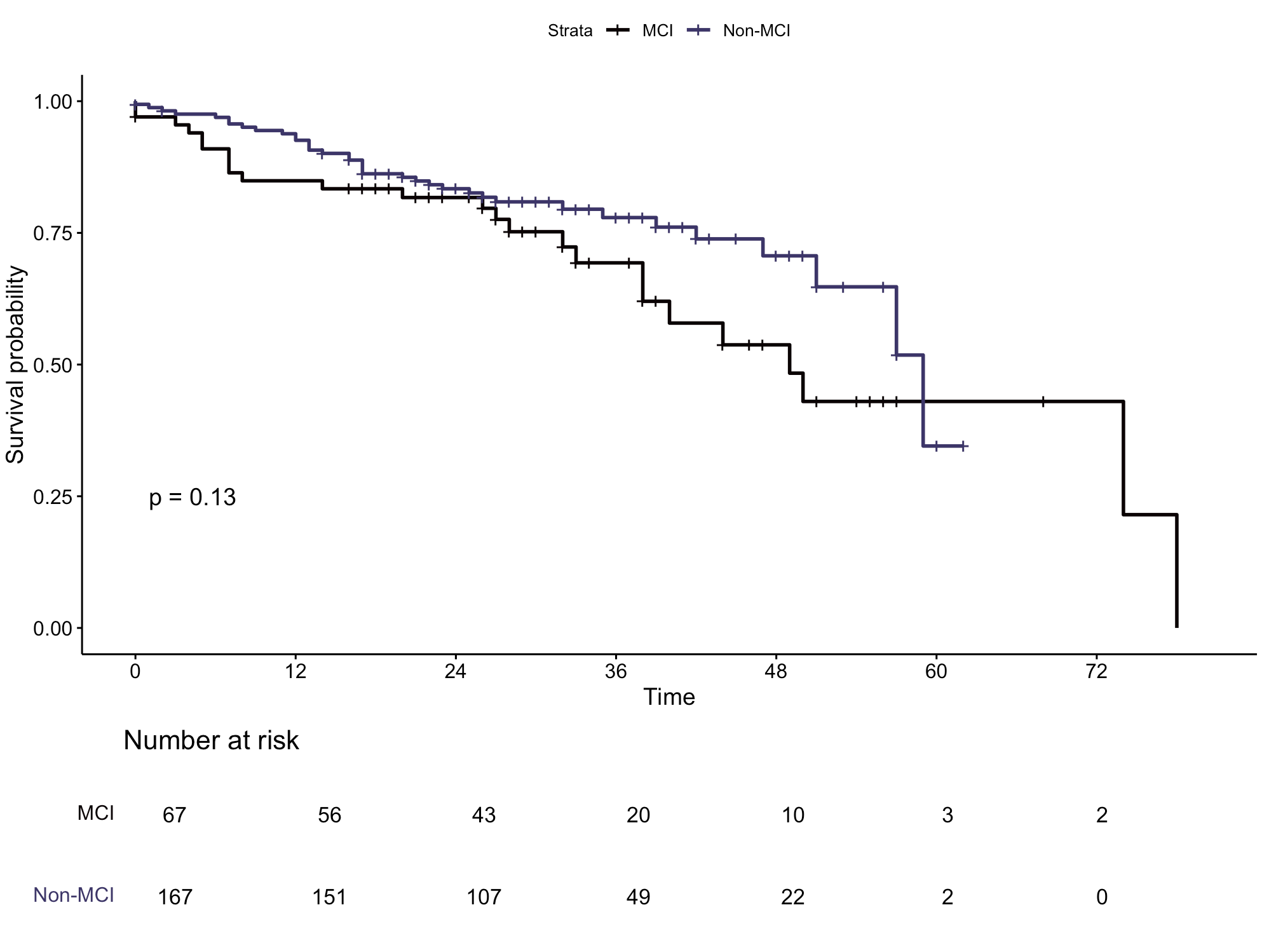


Figure S3: Kaplan-Meier curves demonstrating 12-months survival of patients with and without multichamber impairment (MCI).
